# Mobile Health Technology Interventions for Suicide Prevention: Protocol for a Systematic Review and Meta-Analysis

**DOI:** 10.2196/resprot.8635

**Published:** 2018-01-26

**Authors:** Ruth Melia, Kady Francis, Jim Duggan, John Bogue, Mary O'Sullivan, Derek Chambers, Karen Young

**Affiliations:** ^1^ Psychology Department Health Service Executive Mid-West Ennis Ireland; ^2^ Psychology Department Health Service Executive West Roscommon Ireland; ^3^ College of Engineering and Informatics National University of Ireland Galway Galway Ireland; ^4^ School of Psychology National University of Ireland Galway Galway Ireland; ^5^ Suicide Prevention Resource Office Health Service Executive West Galway Ireland; ^6^ ReachOut Ireland Dublin Ireland

**Keywords:** mHealth, suicide prevention, systematic review, meta-analysis

## Abstract

**Background:**

Previous research has reported that two of the major barriers to help-seeking for individuals at risk of suicide are stigma and geographical isolation. Mobile technology offers a potential means of delivering evidence-based interventions with greater specificity to the individual, and at the time that it is needed. Despite documented motivation by at-risk individuals to use mobile technology to track mental health and to support psychological interventions, there is a shortfall of outcomes data on the efficacy of mobile health (mHealth) technology on suicide-specific outcomes.

**Objective:**

The objective of this study is to develop a protocol for a systematic review and meta-analysis that aims to evaluate the effectiveness of mobile technology-based interventions for suicide prevention.

**Methods:**

The search includes the Cochrane Central Register of Controlled Trials (CENTRAL: The Cochrane Library), MEDLINE, Embase, PsycINFO, CRESP and relevant sources of gray literature. Studies that have evaluated psychological or nonpsychological interventions delivered via mobile computing and communication technology, and have suicidality as an outcome measure will be included. Two authors will independently extract data and assess the study suitability in accordance with the Cochrane Collaboration Risk of Bias Tool. Studies will be included if they measure at least one suicide outcome variable (ie, suicidal ideation, suicidal intent, nonsuicidal self-injurious behavior, suicidal behavior). Secondary outcomes will be measures of symptoms of depression. Where studies are sufficiently homogenous and reported outcomes are amenable for pooled synthesis, meta-analysis will be performed. A narrative synthesis will be conducted if the data is unsuitable for a meta-analysis.

**Results:**

The review is in progress, with findings expected by summer 2018.

**Conclusions:**

To date, evaluations of mobile technology-based interventions in suicide prevention have focused on evaluating content as opposed to efficacy. Indeed, previous research has identified mobile applications that appear to present harmful content. The current review will address a gap in the literature by evaluating the efficacy of stand-alone mobile technology tools in suicide prevention. It is imperative that research identifies the evidence base for such tools in suicide prevention in order to inform policy, guide clinical practice, inform users and focus future research.

**Trial Registration:**

PROSPERO International Prospective Register of Systematic Reviews CRD42017072899; https:// www.crd.york.ac.uk/prospero/display_record.asp?ID=CRD42017072899  (Archived by WebCite at http://www.webcitation.org/ 6tZAj0yqJ)

## Introduction

### Suicide Prevention

Over 800,000 people die due to suicide every year globally, accounting for 1.4% of all deaths worldwide [1]. Suicide occurs regardless of age, and was the second leading cause of death among 15-29 year olds worldwide in 2012. In addition, it is estimated that 25 suicide attempts (100-200 for youth) occur for every death by suicide [2], resulting in more than 400,000 emergency department visits annually in the United States [3]. Suicidal behavior is the result of a complex interaction of psychiatric, psychological, social, and cultural factors [4,5,6]. Prospective studies have attempted to predict which individuals will attempt or die by suicide [7], and a diverse range of risk factors that correlate with suicidal behavior have been proposed to support the identification of those at elevated risk, such as sleep disturbances [8], emotion regulation deficits [9], family history of suicide [10], and chronic pain and illness [11].

Franklin et al [12], in a recent meta-analysis of studies that have attempted to longitudinally predict suicidal thoughts or behavior-related outcomes, found that prediction was only slightly better than chance for all outcomes, and highlighted several fundamental changes needed in future research. They point towards the proliferation of mobile technologies as a means to capture large data sets and to support the expansion of the research-base from a focus on risk *factors* to risk *algorithms*. Furthermore, Kristoufek et al [13], in an attempt to improve the accuracy of suicide estimates, found that estimates drawing on Google search data are significantly better than estimates using previous suicide data alone. Specifically, they found that a greater number of searches for the term “depression” is related to fewer suicides, whereas a greater number of searches for the term “suicide” is related to more suicides.

In parallel, suicide researchers have argued that the Ecological Momentary Assessment (EMA)—high frequency data collection in an individual’s usual environment—provides the potential for the development of a temporal, individualized prediction of risk states. Thompson et al [14] tested the ability of EMA to predict individual symptom change in suicidal ideation in a sample of 35 adults diagnosed with interepisode bipolar disorder. The results showed that EMA with Functional Linear Modeling substantially increased the accuracy of predicting study-emergent suicide ideation. By employing measures of negative and positive effects, cross-validated predictions attained 88% sensitivity with 95% specificity for elevated suicidal ideation one week prior to in-person clinician assessment. Such findings indicate that EMA data could sensitively detect the warning signs of suicidal ideation to facilitate improved suicide risk assessment and the timely delivery of preventative interventions [14]. Advances in mobile technologies provide potential opportunities to operationalize EMA research to support the sensitive and timely identification of those at risk of suicide.

### mHealth and Suicide Prevention

Mobile Health (mHealth) is a component of electronic health (eHealth). The Global Observatory for eHealth defines mHealth as “medical and public health practice supported by mobile devices, such as mobile phones, patient monitoring devices, personal digital assistants (PDAs), and other wireless devices” [15]. According to the World Health Organization (WHO), “mHealth involves the use and capitalization on a mobile phone’s core utility of voice and short messaging service as well as more complex functionalities and applications including general packet radio service, third and fourth generation mobile telecommunications (3G and 4G systems), global positioning system, and Bluetooth technology” [15].

Mobile devices offer a potentially powerful means of delivering evidence-based interventions with greater specificity to the individual and at the time when the intervention is needed. mHealth programs and interventions use mobile technology for a range of functions, from data collection tools for health care professionals and clinical decision support systems to supporting health behavior change and disease management by patients in the community. Two of the major barriers to help-seeking for individuals at risk of suicide are stigma and geographical isolation [16]. Recent advances in mobile health technology could address these main barriers by directing individuals at risk of suicide, who would not otherwise seek help, to appropriate evidence-based online programs or traditional mental health services [17]. The use of digital technology has been found to be beneficial in the delivery of Web-based suicide prevention interventions [18]. Furthermore, a survey in a psychiatric out-patient setting reported that 69% of respondents and 80% of those aged 45 years or younger indicated a desire to use a mobile application to track their mental health [19]. Brathwaite et al [20], amongst other researchers, have begun to validate machine learning algorithms for social networking data against established measures of suicidality.

Despite the motivation to use mHealth technologies for these purposes, there is a lack of outcomes data on the efficacy of mHealth interventions on suicidal behavior. In 2014, Christensen et al [16] conducted literature review on eHealth and suicide, which involved reviewing the effectiveness of eHealth interventions for suicidal thoughts. The majority of eHealth interventions identified in their search were Web-based as opposed to mobile-based. The researchers concluded that there is some evidence to suggest that suicide interventions via the Web may be effective, but only if they target suicidal content specifically, as opposed to the associated symptoms of depression through cognitive behavioral therapy. Given recent developments in technology, particularly in the area of mHealth technology, there is a need to explore the current research on this subject as it relates to suicide prevention.

Donker et al [21] found that mental health apps evaluated in randomized controlled trials were not publicly available, while those with no research evidence were. Larsen et al [22] conducted a comprehensive content review of currently-available smartphone tools for suicide prevention and reported a lack of comprehensive evidence-based support for the mobile apps evaluated. In addition, mobile apps that presented harmful content were also identified [21]. Perry et al [23] conducted a systematic review of online and mobile psychosocial suicide prevention interventions for adolescents and young adults. The researchers searched four major psychological databases for interventions that explicitly targeted suicidality using a mobile, computer, or Web-based app for individuals aged between 12 and 25 years. However, only one study met the authors’ inclusion criteria and therefore, a meta-analysis could not be conducted. Building on the work of Perry and colleagues [23], the current review will aim to address the disparity that exists between the availability of mHealth suicide prevention tools and clinical trial data. The current review will broaden the search strategy to include unpublished studies and ongoing trials of mhealth technology for suicide prevention as previous reviews of digital interventions for suicide prevention have identified a very limited number of mobile apps [23,24] In addition, studies will not be excluded based on participants’ age.

### The Importance of This Review

While there has been a rise in the number of mobile technology tools for suicide prevention, there is a dearth of research evaluating the efficacy, and relative strengths and weaknesses of this modality. Research evaluating the content and usability of such tools has been undertaken, but the need to examine outcomes is necessary, particularly given that many of these tools are currently available and utilized. From the perspectives of researchers, policy developers, health care providers, and suicide prevention mobile app users, it is imminently important to assess the effectiveness of this method and to highlight its most efficacious components.

The current review will build upon the systematic assessment of smartphone tools for suicide prevention carried out by Larsen et al [22], which examined the concordance of features in publicly available mobile applications with current scientific evidence for effective suicide prevention strategies. Systematic review methodology was used to screen and assess app content. Therefore, the aim of the current research is to further advance this research by focusing on the efficacy of interventions delivered via mobile technologies for suicide-specific outcomes.

The objective of this review is to examine the effectiveness of stand-alone mobile technology tools in reducing suicide-specific outcomes.

## Methods

### Eligibility Criteria

This protocol has been developed in line with the Preferred Reporting Items for Systematic Review and Meta-Analysis Protocols statement [25]. The systematic review and meta-analysis will be conducted and reported in accordance with the Preferred Reporting Items for Systematic Reviews and Meta-Analyses (PRISMA) statement [26] and has been registered with the International Prospective Register of Systematic Reviews (PROSPERO) database (registration number: CRD42017072899). In accordance with the PRISMA checklist recommendations, this review will use the Participants, Interventions, Comparisons, Outcome(s) process for framing and reporting the review criteria, and the study design of the included studies will be reported.

### The Review Team

The review team will manage and conduct the review, and will have experience in systematic review methods, information retrieval, and statistics. A minimum of two researchers will be involved to minimize bias and error at all stages of the review.

In addition to the review team, an advisory group will be consulted at various stages, including health care professionals, experts-by-experience, and experts in research methods.

### Types of Studies

This review is a systematic review of mHealth technology interventions for suicide prevention. As in previous research, which reviewed digital interventions for suicidal ideation and self-harm [24], types of studies included will be randomized controlled trials (RCTs), pseudo-RCTs, and observational pretest/posttest designs that evaluate the effectiveness of mHealth technology in suicide prevention. Due to the expectation of a limited number of publications available, the search strategy will not be restricted to RCTs and will include both published and unpublished trials. Studies will be included if the full report is accessible in English. Only studies that evaluated mobile tools that related specifically to suicide prevention or where suicidality is explicitly mentioned will be included.

### Types of Participants

Participants will be individuals at risk of suicide who took part in a suicide prevention intervention via mHealth technology. No restriction will be placed on the age or gender of participants included in the studies reviewed. Mobile health technology represents a modality that is accessible across the lifespan. However, the review will note the age of participants included in each study where this information is available and use this information to draw conclusions regarding the efficacy of this method for specific age groups.

### Types of Interventions

Included studies must report on a suicide prevention intervention delivered via mHealth technology. That is, interventions must aim to reduce suicide risk by employing mobile communication or mobile computing technology. Studies must report the effects of the intervention on a suicide-specific outcome. The review will include studies with psychological and nonpsychological interventions (eg, psycho-education, diaries, mood monitors, and self-management programs). As defined by Slattery et al [27] in a protocol for a systematic review on eHealth interventions for chronic pain, psychological treatments are those that explicitly deliver a psychological component (eg, psychotherapy for suicidal thoughts). Studies will be included regardless of treatment intensity or duration. Studies reporting on stand-alone mobile interventions only will be included.

### Types of Outcome Measures

Included studies must have at least one suicide-specific outcome as a primary outcome. This will include suicidal behavior, nonsuicidal self-injurious behavior, suicidal ideation, and suicidal intent. Secondary outcomes will be symptoms of depression, as measured using administered or self-reported scales.

### Search Strategy

All databases will be searched from their start date. Studies will be included if a full-text paper is made available in English, either through databases or through contact with the study authors. The following databases will be searched: MEDLINE, Embase, PsycINFO, CENTRAL (Cochrane Library), and Centre for Research Excellence in Suicide Prevention. The same search strategy will be used for each database; however, appropriate changes will be made to accommodate the different interfaces. Details of the search strategy are provided in Textbox 1. Medical Subject Headings or equivalent and text word terms will be used.

Clinical trial registries will be searched to identify completed and in-progress trials. This will include ClinicalTrials.gov (), the metaRegister of controlled trials () and the World Health Organization International Clinical Trials Registry Platform (). Gray literature will be searched using the OpenGrey database (), which includes technical or research reports, doctoral dissertations, and conference papers from the last 5 years.

The reference lists of relevant systematic reviews and of included studies will be searched in order to identify additional studies that may be relevant.

### Selection of Studies

Studies that are identified by our search strategy will be managed using Endnote X8 [28]. Members of the research team will initially screen the titles and abstracts of publications for duplicates. Members of the research team will then screen for any studies that are not relevant to the review and will exclude them by adding them to a global exclusion folder. All remaining publications will be retrieved for further scrutiny. Two reviewers will independently assess the full text of the remaining studies for inclusion in line with the exclusion criteria. Papers that do not meet the inclusion criteria will be systematically excluded via the exclusion categories and the reason for exclusion will be recorded. Disagreements between reviewers will be discussed until resolved; in the event a resolution cannot be reached, a third reviewer will arbitrate. A record will be kept of all articles excluded at this stage. A PRISMA flow chart will be created to graphically depict the inclusion and exclusion of studies.

### Data Extraction and Management

A data extraction form will be created prior to data extraction. Data will be extracted independently by one reviewer and verified by another reviewer using a customized form, which will be piloted prior to use. The finalized data will be entered into RevMan 5.3 *.* Where the necessary outcome data are unavailable, the study authors will be contacted. The authors will not be blind to the study author, institution or journal. Data will be extracted relevant to the following categories: (i) study population and design; (ii) intervention; and (iii) outcome. Characteristics of table(s) in included studies’ will be created and will include the following information where available:

- Participant characteristics
- Geographic location
- Assessment periods
- Assessment / screening measures
- Description of intervention and comparison interventions
- Primary and secondary outcomes
- Theoretical basis
- Therapeutic content
- Mode of delivery (smartphone application, telephone, text)
- Suicide prevention strategies
- Behavior change techniques
- Control condition
- Intensity and frequency of use
- Treatment engagement (retention and attrition)

### Assessment of Risk of Bias in Included Studies

The reviewers will independently assess risk of bias using the recommended Cochrane Collaboration’s Risk of Bias tool [29] for randomized and pseudo-RCTs. The Risk of Bias in Nonrandomized Studies of Interventions (ROBINS-I) [30] will be used to assess risk of bias for controlled before/after designed studies. The Cochrane Collaboration tool assesses randomization procedures, bias, allocation, outcome assessor, reporting of findings, and losses to follow-up. Studies are then classified having a low, high or unclear risk of bias. The ROBINS-I assesses confounding participant selection, classification of the intervention, departures from the intended intervention, missing data, measurement of outcomes, selection of the reported results, and overall bias. The ROBINS-I classifies studies as being of low, moderate, serious, or critical risk of bias.

**Details of the search terms to be used.**

mobile* OR mobile phone OR cell* or cell phone* OR

mobile health OR m-health OR mhealth OR mobile app* OR mobile technolog* OR text messag* OR smartphone OR personal digital assist* OR PDA OR patient monitoring device OR PMD

suicid* OR suicide gesture OR suicidal behavio* OR suicidal idea* OR suicide attempt OR self-mutilation OR self harm OR self-harm OR self [-] injury OR suicide OR suicidal intent OR deliberate self-harm OR DSH OR deliberate self poisoning OR self cutting OR self-inflicted wound OR deliberate self cutting

### Statistical Methods

In the event that only a small number of studies are identified with a large amount of heterogeneity present, a full narrative review will be undertaken using the “Narrative Synthesis in Systematic Reviews” tool [31].

Where a sufficient number of papers are identified that meet the outlined inclusion criteria, the meta-analysis will be conducted. The level of heterogeneity will also be taken into account when considering the suitability of the data for a meta-analysis.

If deemed appropriate, a meta-analysis will be conducted. RevMan 5.3 will be used for all analyses. For continuous data, we will report the mean differences between groups and the 95 % confidence interval (95 % CI). Where no standard deviations are reported, we will calculate the standard deviation using the methods described in the Cochrane Handbook for Systematic Reviews of Interventions [29]. Where the same outcome is measured using different measurement tools, we will calculate the standardized mean difference and the 95 % CI for continuous data.

It is expected that many different intervention types, participants and comparators will be examined across studies, sufficient to expect that underlying treatment effects would differ between the included studies. Therefore, a random effects meta-analysis model will be used.

#### Assessment of Heterogeneity, Sensitivity and Publication Bias

Statistical heterogeneity will be assessed using χ^2^, I^2^, and T^2^. χ^2^ assesses whether observed differences in results are compatible with chance alone. Statistical heterogeneity will be regarded as substantial if the χ^2^*P*-value is <.01. The I^2^ statistic represents the percentage of the total variation across studies due to heterogeneity. The Cochrane Handbook [32] suggests that an I^2^ value of less than 40% is an unsignificant amount of heterogeneity. T^2^ provides an estimate of the between-study variance in a random effects meta-analysis. A T^2^ value of greater than 1 indicates substantial heterogeneity. Data will be analyzed using RevMan 5.3.

Sensitivity analysis will be conducted by examining whether the exclusion of studies which were identified as having greater risk of bias affects the effect sizes and comparisons between groups.

Publication bias will be assessed using Egger’s test [33] and funnel plots conducted if there are a sufficient number of studies (>10).

#### Subgroup Analyses

The inclusion of RCT’s and nonrandomized observational studies within a single meta-analysis has become increasingly common [24] as relying on data from RCTs alone can lead to knowledge translation bias [34]. The inclusion of results from pretest/posttest

observational studies together with those from RCTs, however, can also lead to over-estimation of the treatment effect size [35]. To address these concerns, RCTs, pseudo-RCTs, and observational pretest/posttest designs will be eligible for inclusion in this review. However, we will not pool data from RCTs together with data from observational studies. Separate subgroup analyses will be conducted by study design to investigate the impact, if any, that study design has on the magnitude of the effect size observed for the included interventions.

## Results

This systematic review is currently underway, with results anticipated by summer 2018. The anticipated findings of this review are likely to inform policy, guide clinical practice, and users, and build on current research in the area of suicide prevention.

## Discussion

### Rationale for This Study

This systematic review will address a significant lack of outcomes research examining the efficacy of mobile technology–based interventions in suicide prevention. The lack of research is pertinent given the recent increase in the development and usage of such tools for this purpose.

This review will be an extension of Larsen et al’s [22] review by systematically assessing smartphone tools for suicide prevention by (a) not restricting the modalities reviewed to smartphone apps and including other mobile technology–delivered interventions; and (b) evaluating efficacy using outcomes research in order to complement their comprehensive assessment of content.

Where data is available, a comparison of mobile technology tools across outcome measures (ie, smartphone applications, text etc) would greatly inform clinicians, developers, policy-makers, and researchers on the most effective modes of delivery.

### Limitations

In this study, a limited number of available studies is expected. Including studies examining a broad range of mobile technology tools generally as opposed to smartphone apps specifically will go some way to addressing this. Similarly, including studies which may have a mental health condition such as depression as their primary focus and which include suicide-specific primary outcomes should allow for all relevant data to be collected.

### Implication of the Review

To the best of the researcher’s knowledge, no such meta-analysis has been reported that examined the effectiveness of mHealth technology interventions, in particular suicide-specific outcomes. This review will provide guidance for further research, valuable information to clinicians, and support the standardization of practice and policy in relation to the use of mobile technology in suicide prevention.

## Abbreviations

eHealth: electronic health
EMA: Ecological Momentary Assessment
mHealth: mobile health
PDA: patient digital assist
PMD: patient monitoring device
PRISMA: Preferred Reporting Items for Systematic Reviews and Meta-Analyses
RCT: randomized controlled trial
ROBINS-I: Risk of Bias in Nonrandomized Studies of Interventions
WHO: World Health Organization
